# The risk of going outside: Amino phospholipids in rheumatoid arthritis

**DOI:** 10.1016/j.jlr.2025.100870

**Published:** 2025-07-29

**Authors:** Nicola Pozzi, David A. Ford

**Affiliations:** Edward A. Doisy Department of Biochemistry and Molecular Biology and Center for Cardiovascular Research, Saint Louis University School of Medicine, St. Louis, MO, USA

Rheumatoid arthritis (RA) is an autoimmune disease that affects approximately 1.5 million people in the United States and is 2–3 times more likely to occur in women than in men (1). It primarily targets the joints but also contributes to interstitial lung injury, vasculitis, and cardiovascular disease. RA most commonly develops in adults between the ages of 30 and 60, posing a substantial health and economic burden.

A critical driver of RA is immune system dysfunction, which triggers the production of autoantibodies and disrupts cytokine signaling pathways. This synergistic disturbance fosters a highly pro-inflammatory environment where lipid mediators assume a central role. The interplay between immune dysregulation and increased inflammatory lipid production underpins current RA therapies, aimed at mitigating inflammation and modulating immune activity. These include nonsteroidal anti-inflammatory drugs (NSAIDs), biologics targeting TNF-α, IL-6, and RANKL, as well as B-cell-depleting agents that reduce autoantibody synthesis. Despite these advances, treatment efficacy remains suboptimal for many patients and is accompanied by significant risks, particularly increased susceptibility to infections resulting from broad immunosuppression (1).

In addition to systemic inflammation, venous thromboembolic events (VTEs), including deep vein thrombosis and pulmonary embolism, are significant and potentially fatal complications of RA. All three components of Virchow's triad (endothelial dysfunction, blood flow stasis, and hypercoagulability) are implicated. Cellular contributors include endothelial cells, platelets, and white blood cells, although the precise role of each remains unclear.

Thrombus formation requires thrombin, the enzyme that catalyzes the conversion of soluble fibrinogen into insoluble fibrin (2). Thrombin is generated from prothrombin by the prothrombinase complex, which requires calcium ions and anionic phospholipid surfaces, most notably phosphatidylserine (PS) (2, 3). Other amino phospholipids, such as phosphatidylethanolamine (PE), support PS's role in thrombin production (4). While endothelial cells are central to prothrombinase assembly during primary hemostasis, platelets and white blood cells also contribute, especially in pathogenic contexts and in the absence of vascular injury, by providing suitable surfaces for prothrombinase assembly, either directly or via the release of prothrombotic extracellular vesicles (EVs). In patients with RA, the role of platelets, white blood cells, and EVs in thrombin generation has been considered. Early studies reported elevated EV levels in the plasma of patients with RA, compared to healthy controls (5, 6), suggesting a prothrombotic phenotype. However, the detailed chemical composition and molecular species of these EVs remained unclear.

In this issue of JLR, the O'Donnell group presents, for the first time, the anionic phospholipid composition of platelets, white blood cells, and EVs from RA patients and their contribution to thrombosis risk (7). Among these, EVs emerged as key contributors. Not only did they confirm that plasma EVs are elevated in patients with RA (Fig. 1A), consistent with previous findings, but they also demonstrated that higher EV levels correlate with increased thrombin generation in RA plasma, showing a strong positive correlation with thrombin-antithrombin (TAT) complexes and thrombin generation in vitro. In contrast, platelets and white blood cells did not increase thrombin generation in their assays.

**Fig. 1:**
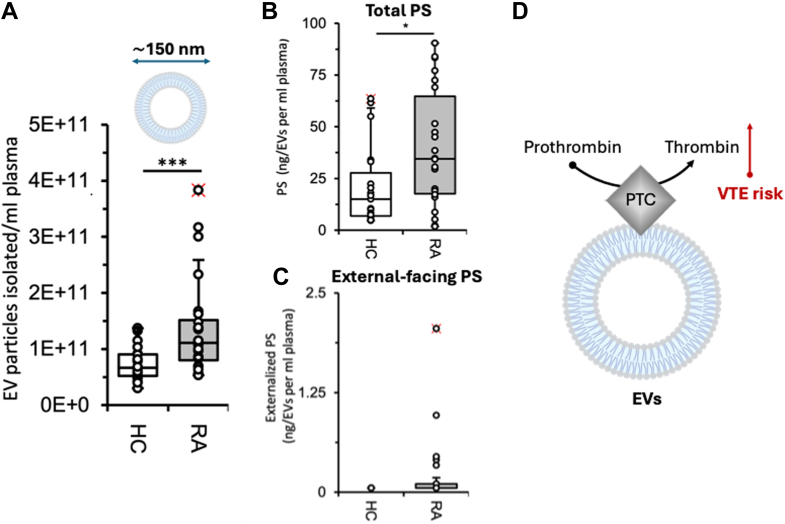
**Extracellular vesicles (EVs) increase thrombotic risk in rheumatic arthritis (RA).** A: Levels of EVs are elevated in RA patients compared to healthy controls (HC). These EVs contain more (B) phosphatidylserine (PS), some of which are (C) externally facing. D: External-facing PS on EVs stimulates prothrombinase support, assembly of prothrombinase complex (PTC), and promotes thrombin generation, thereby increasing the risk of venous thromboembolism (VTE). Panels A, B, and C are from Figs. 1E, 1M, and 1K, respectively, of the work by Costa D. *et al.* (7).

Given the central role of PS in thrombin generation, the authors performed rigorous lipidomic analyses to determine both the total phospholipid content and the sidedness of EVs (ie, which phospholipids are exposed on the outer surface) using biotin derivatization followed by LC/MS. This is critical, as only externally facing phospholipids interact with plasma proteins and coagulation factors. While similar levels of PE were observed in EVs from both patients with RA and healthy controls, PS was significantly enriched in RA EVs (Fig. 1B). Notably, externally facing 18:0–20:4 and 18:0–18:1 PS species were detected in RA EVs but not in those from healthy controls (Fig. 1C). These findings support the hypothesis that EVs containing external PS species contribute to thrombin generation in RA plasma, reinforcing the proinflammatory and prothrombotic phenotype (Fig. 1D).

Interestingly, the anionic phospholipid profile of RA EVs resembles that seen in patients with atherosclerotic cardiovascular disease (8), suggesting a shared mechanism of EV production. This raises the possibility that similar EV-mediated thrombotic mechanisms may be active in other autoimmune diseases characterized by platelet and white blood cell activation and elevated EV levels, such as systemic lupus erythematosus (SLE) (6) and antiphospholipid syndrome (APS) (9). For example, women with SLE aged 35–44 have a 50-fold increased risk of myocardial infarction, a risk not explained by traditional Framingham factors (10, 11). APS is associated with a high risk of cardiovascular events, driven by antiphospholipid antibodies that target anionic phospholipids as well as plasma proteins bound to anionic phospholipids (12). Measuring the levels of externally facing PS and the prothrombotic activity of EVs in these patients may help identify individuals at elevated risk of thrombosis and cardiovascular complications, pointing to a broader paradigm of autoimmune-driven vascular disease. Understanding the efficiency and molecular mechanisms by which PS-enriched EVs support the conversion of prothrombin to thrombin is a critical area of research with high therapeutic potential. If EVs are confirmed to significantly contribute to thrombosis risk, strategies such as EV depletion or surface masking could be explored to reduce thrombin generation. The work by the O'Donnell group adds important mechanistic detail to our understanding of RA-associated thrombosis. The methodologies introduced in this study provide a valuable framework for future investigations into the vascular complications of RA and related autoimmune diseases.

## Data availability

All data presented are contained within the manuscript.

## Conflict of interest

The authors declare that they have no conflicts of interest with the contents of this article.
